# Estimating the age of the p.Cys433Arg variant in the *MYOC* gene in patients with primary open-angle glaucoma

**DOI:** 10.1371/journal.pone.0207409

**Published:** 2018-11-16

**Authors:** Ana Maria Marques, Galina Ananina, Vital Paulino Costa, José Paulo Cabral de Vasconcellos, Mônica Barbosa de Melo

**Affiliations:** 1 Center for Molecular Biology and Genetic Engineering (CBMEG), University of Campinas (UNICAMP), Campinas, São Paulo, Brazil; 2 Department of Ophthalmology and Otorhinolaryngology, School of Medical Sciences, University of Campinas (UNICAMP), Campinas, São Paulo, Brazil; Ohio State University Wexner Medical Center, UNITED STATES

## Abstract

The aim of this study was to estimate the age of the Cys433Arg (c.1297T>C, p.Cys433Arg) variant by comparing the genotypes of individuals affected and not affected by primary open angle glaucoma juvenile onset (JOAG). Our sample consisted of 35 JOAG-affected individuals from three families, 16 unrelated patients with the *MYOC* p.Cys433Arg variant and 16 unaffected individuals. Genomic DNA was amplified by PCR; nine short tandem repeats were genotyped through automated electrophoresis and three single nucleotide polymorphisms through Sanger sequencing. The determination of haplotypes was performed using Arlequin software and age estimation was performed using DMLE+ 2.3 and BDMC21 softwares. Four markers constituted the haplotypes associated with the p.Cys433Arg variant. The software DMLE+2.3 predicted an age of 43 generations for this variant with a 95% confidence interval ranging from 28 to 76 generations (560–1520 years) and BDMC21 predicted an age of 59 generations (1180 years) (95% CI: 40 to 100).

## Introduction

Glaucoma is the leading cause of irreversible blindness [1], affecting more than 60 million people worldwide and projected to reach 76 million by 2020 [2]. It is characterized by degeneration of retinal ganglion cells, loss of neural tissue from the optic nerve head and correspondent visual field defect [3].

Primary open-angle glaucoma [POAG] is the most common form of the disease with a complex inheritance pattern [4,5]. The main risk factor for POAG is elevated intraocular pressure (IOP), in addition to positive family history, advanced age and black ethnicity [6,7]. Primary open angle glaucoma juvenile onset (JOAG) is a subtype of POAG characterized by an early onset (3 to 40 years of age), usually following an autosomal dominant inheritance pattern and more aggressive clinical presentation, which frequently requires surgical intervention [8,9].

Several *loci* have already been associated with POAG, and the most reproducible and representative is the *GLC1A locus*, where the *MYOC* gene was subsequently identified [10,11]. The *MYOC* gene is composed by three exons with 606, 126 and 718 bp, respectively, and encodes the myocillin protein which is expressed in a variety of ocular tissues including the trabecular meshwork, cornea, retina and ciliary body [12–14]. It has been reported that 8% to 30% of patients with JOAG and 2 to 4% of patients with POAG have disease causing variants in the *MYOC* gene [15,16], evidencing the importance of this gene to glaucoma’s etiology.

Vasconcellos et al carried out an investigation in 25 unrelated JOAG patients from Brazil and first reported the *MYOC* p.Cys433Arg (c.1297T>C) variant, which is the replacement of a cysteine for an arginine at codon 433 in the exon 3 of the *MYOC* gene [17]. This variant is involved in the intracellular sequestration (in the endoplasmic reticulum of trabecular meshwork cells) of insoluble aggregates of mutant myocilin, which represents the same pattern of other disease-causing variants. This condition may lead to an increase in oxidative stress that may facilitate the death of these cells and thus impair the output of aqueous humor, ultimately leading to increased IOP [18–20].

This variant was found only in Brazilian patients and has never been reported in other countries [17,21]. Searches performed in the Brazilian databases BIPMed [http://bipmed.iqm.unicamp.br/] and ABraOM (http://abraom.ib.usp.br/) and in international databases ExAC Browser (http://exac.broadinstitute.org/) and 1000 genomes (http://www.internationalgenome.org/) found no registration of this variant. The few studies about this variant reveal high penetrance in patients with JOAG, ranging from 28 to 100% depending on the age of the patient [17,21,22]. Case-control studies report a high prevalence of the p.Cys433Arg variant in the state of São Paulo; approximately 30% of unrelated JOAG patients that harbor variants in the *MYOC* gene have this variant [23].

In the first study, which reported the p.Cys433Arg variant, Vasconcellos and colleagues analyzed four microsatellite markers in unrelated patients with JOAG who carried this variant and showed that six patients exhibited the same haplotype, indicating that these patients inherited the variant from a common ancestor [17]. Based on this fact, the aim of this study was to estimate the age of the p.Cys433Arg variant, contributing to the understanding of the demographic history of this variant in the Brazilian population, and to estimate the population at risk to harbor it.

## Methods

### Patients

**Fig 1:**
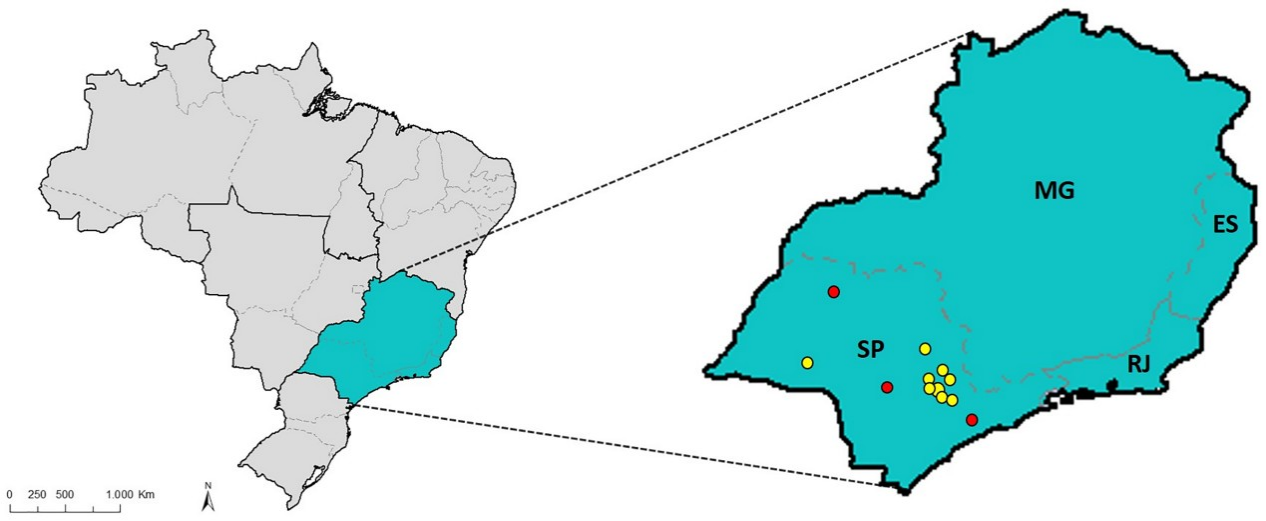
Map of Brazil emphasizing the Southeast region. Southeast region of Brazil formed by the states of São Paulo (SP), Minas Gerais (MG), Rio de Janeiro (RJ) and Espírito Santo (ES). The red points represent the hometowns of the families (Botucatu, Tanabi and Mogi das Cruzes). Yellow points represent the hometowns of the unrelated patients (Campinas, Limeira, Americana, Mogi Guaçu, Jundiaí, Pirassununga, Itapira and Paraguaçu Paulista).

This study consisted of 51 individuals, of whom 35 came from three different families and 16 were unrelated individuals, all affected by JOAG, with age at diagnosis less than 40 years-old and harboring the p.Cys433Arg variant in the *MYOC* gene. The control group consisted of 32 unrelated individuals unaffected by the disease and above 50 years of age. Both patients and controls are from the State of São Paulo, Southeast region of Brazil (Fig 1). In terms of ethnicity most of the patients and controls analyzed in this study were classified by the observers as “pardos”, which is a term used in the Brazilian census by the Brazilian Institute of Geography and Statistics (Instituto Brasileiro de Geografia e Estatística—IBGE) to refer to individuals of mixed race origin, as a result of a miscegenation among European, African and Amerindian descendants. The study was approved by the Research Ethics Committee of the Faculty of Medical Sciences, University of Campinas (CAAE: 49120615.4.0000.5404). Written informed consent was obtained from all participants.

A comprehensive ophthalmological examination, including visual acuity measurement, slit-lamp biomicroscopy, Goldmann applanation tonometry, gonioscopy and optic disc evaluation (with the pupils dilated and using a 78-diopter lens) were performed. The definition of JOAG was based on structural and functional changes observed in the optic disc and visual field evaluations, respectively, as well as an open angle at gonioscopy. The optic disc was considered to have glaucoma if it exhibited at least two of the following signs: thinning of the neuroretinal rim, haemorrhage, notch, cup/disc ratio >0.7, or asymmetry of the cup/disc ratio >0.2. Glaucomatous visual field defect was defined as a pattern standard deviation outside of 95% of the normal limits or a glaucoma hemifield test result outside of the 99% limit and a correspondent cluster of points with p < 0.05% in the pattern deviation graphic.

The participants had their biological materials collected at the Glaucoma Service, Clinical Hospital of the University of Campinas and were identified as having the *MYOC* p.Cys433Arg variant in previous studies [17,20,22,23].

### Genotyping

Nine short tandem repeats (STRs) and three single nucleotide polymorphisms (SNPs) have been selected in a 5Mb region around the p.Cys433Arg variant in the *MYOC* gene (see S1 Table). The PRIMER3 (http://primer3.ut.ee/) software was employed to predict the primers. The forward primers of the STRs were labeled with HEX or FAM fluorophores at the 5' end in order to assemble multiplex reactions with three markers on each. The fragments of interest were amplified by PCR and confirmed in 1.5% agarose gel electrophoresis. The descriptions of the primers are shown in Tables 1 and 2.

**Table 1:** Primers used in the polymerase chain reaction (PCR) for single nucleotide polymorphisms (SNPs) genotyping.

Molecular Marker	Primer sequences (5’-3’)	M/m	Annealing temperatures (°C)	Product sizes (bp)
**rs6133 (F)**	ACTGCTGTCCATTGTCCTGA	C/A	59	197
**rs6133 (R)**	ACAGGCATAGCATCACTTCCT			
**rs2266782 (F)**	TGCTGTAATGGTTTGTTCCGG	G/A	59	238
**rs2266782 (R)**	GCGACCTTGTGAATAGATGCA			
**rs2266780 (F)**	TGGCATTGTGTCCGTAAAGC	A/G	59	237
**rs2266780 (R)**	TGGACAAAGCCAATCACTGC			

M/m denotes major allele/minor allele

**Table 2:** Primers used in the polymerase chain reaction (PCR) for short tandem repeats (STRs) genotyping.

Molecular marker	Primer sequences (5’-3’)	Fluorophores	Annealing temperatures (°C)	Product sizes (bp)	Repetition unit
**rs3219828 (F)**	TGCCTCAGTTCATTCCCCAT	FAM	57	155–177	(CA)
**rs3219828 (R)**	ACTCAATGTTCAAGTTCTCCCTG				
**rs3221612 (F)**	GCCCAGCAAAGAGACCTTCA	HEX	57	179–250	(TG)
**rs3221612 (R)**	CTTCGAACACGTCTTCCCAC				
**rs3219958 (D1S218) (F)**	TGTAAAAGCAAACTGTAGACGAT	FAM	57	266–286	(TG)
**rs3219958 (D1S218) (R)**	TTTATGTTATCACCAAGGCTTCT				
**rs2234708 (F)**	ACATCCCAATTTATGCACCCA	FAM	59	190–207	(AC)
**rs2234708 (R)**	CACAGTGCAGGTTCTCAATGA				
**D1S2815 (F)**	CTGACATGGAATACCTCTATGATGC	HEX	59	210–237	(CA)
**D1S2815 (R)**	CTCCAAATCTAGTCACACTGGAAG				
**rs3223566 (D1S2790) (F)**	GAAGCCTTTCTGTGCTAGCC	FAM	59	243–253	(TG)
**rs3223566 (D1S2790) (R)**	GCACAATGGCCAATGTTTTCA				
**D1S1165 (F)**	AGGCAACAGAGTGAGACTC	FAM	58	117–210	(GGAA)
**D1S1165 (R)**	CTTCCATAGCTGATACTGCT				
**rs3220994 (F)**	GCGCTTGTAATCCCAGCTAC	HEX	58	196–224	(CA)
**rs3220994 (R)**	AGTTGATGGGTAGGGGTGTG				
**rs3220452 (F)**	CTCTCTACCACTTGAATTCCTGT	FAM	62	215–249	(TG)
**rs3220452 (R)**	GAGCCACCACTCCAGTTTGA				

SNPs were sequenced by Sanger method in the ABI PRISM 3500 DNA Analyzer (Applied Biosystems, Foster City, CA, USA) and the sequences obtained were analyzed by the FinchTV software (Geopiza, Seattle, WA, USA). The STRs PCR products were genotyped by automated electrophoresis in the same equipment and analyzed with Geneious version 10.0 software (http://www.geneious.com) [24] (see S2 Table for genotypes).

To determine the haplotypes of unrelated individuals we used the Arlequin v3.5 software [25] and for the families’ individuals the haplotypes were determined by pedigree analysis.

### Age estimation

The age of the variant was estimated by the DMLE+ 2.3 [26] and BDMC21 [27] softwares. DMLE+ 2.3 allows Bayesian inference to calculate the age of the variant (in generations) based on the linkage disequilibrium observed in multiple genetic markers. This software uses the Markov Chain Monte Carlo algorithm (MCMC) which is based on massive random simulations, to calculate the probability of the occurrence of each event. For this study, four million repetitions have been used to obtain the results.

To estimate the age of the variant using DMLE+ 2.3, it was necessary to determine the proportion of mutant chromosomes (0.0294). This parameter was calculated based on the frequency of JOAG in the Brazilian population (150,000 affected people—http://www.who.int), the frequency of the variant in the affected individuals (30%) [17,21–23] and the size of the population in the Southeast region of Brazil, the region where the variant has been reported so far (80,350,000 inhabitants—http://www.ibge.gov.br/). Using this data, we were also able to calculate the number of mutant chromosomes in the population of the Southeast region of Brazil. These calculations were performed using the world population (7.465.000.000 inhabitants—http://www.ibge.gov.br/), the population affected by JOAG and the frequency of the variant in people affected by JOAG. The genetic distances between the markers and between the markers and the variant were extrapolated from physical distances using the Map Interpolator software from The Rutgers Combined Linkage—Physical Map [28]. The average Brazilian population growth rate for the last 300 years was taken as 0.095 (http://www.ibge.gov.br/home/). In addition to these data, haplotypes containing and not containing the variant were added to the software input file. BDMC21 is a maximum likelihood method based on the extent of genetic variation within an allelic class and the allele frequency [27, 29]. To calculate the age of the variant using BDMC21, we applied the same Brazilian population growth rate and the proportion of mutant chromosomes of DMLE+ 2.3. In this software we used ten thousand repetitions of MCMC.

Labuda et al. (1996, 1997) [30–32] suggested that the age of the variant can be underestimated when the study population is growing because the genetic clock becomes slower than expected. Due to recombination events, LD has been decreasing over the time from the founding event until now and the estimation method takes into account the LD measure and recombination rates. However, in growing populations, in the first generations, immediately after the founding event, recombination occurs less than expected. Therefore, a possible approach to this problem is to define the genetic clock through the Luria-Delbrüch correction [33,34]. For the parameters of the calculation we used the population growth rate (0.095) and the recombination rate (equivalent to genetic distance—0.0076 cM) between markers flanking the haplotype (among markers rs3219828 and rs2234708).

## Results

### Population analysis of SNPs

Population analysis of the markers showed that, in the sample of unrelated individuals, the rs6133 SNP was monomorphic, presenting only the C allele. The marker rs2266782 exhibited the G allele more frequently than the A allele, whereas for marker rs2266780, the A allele was identified as the more frequent.

### Haplotypes and age of variant

**Fig 2:**
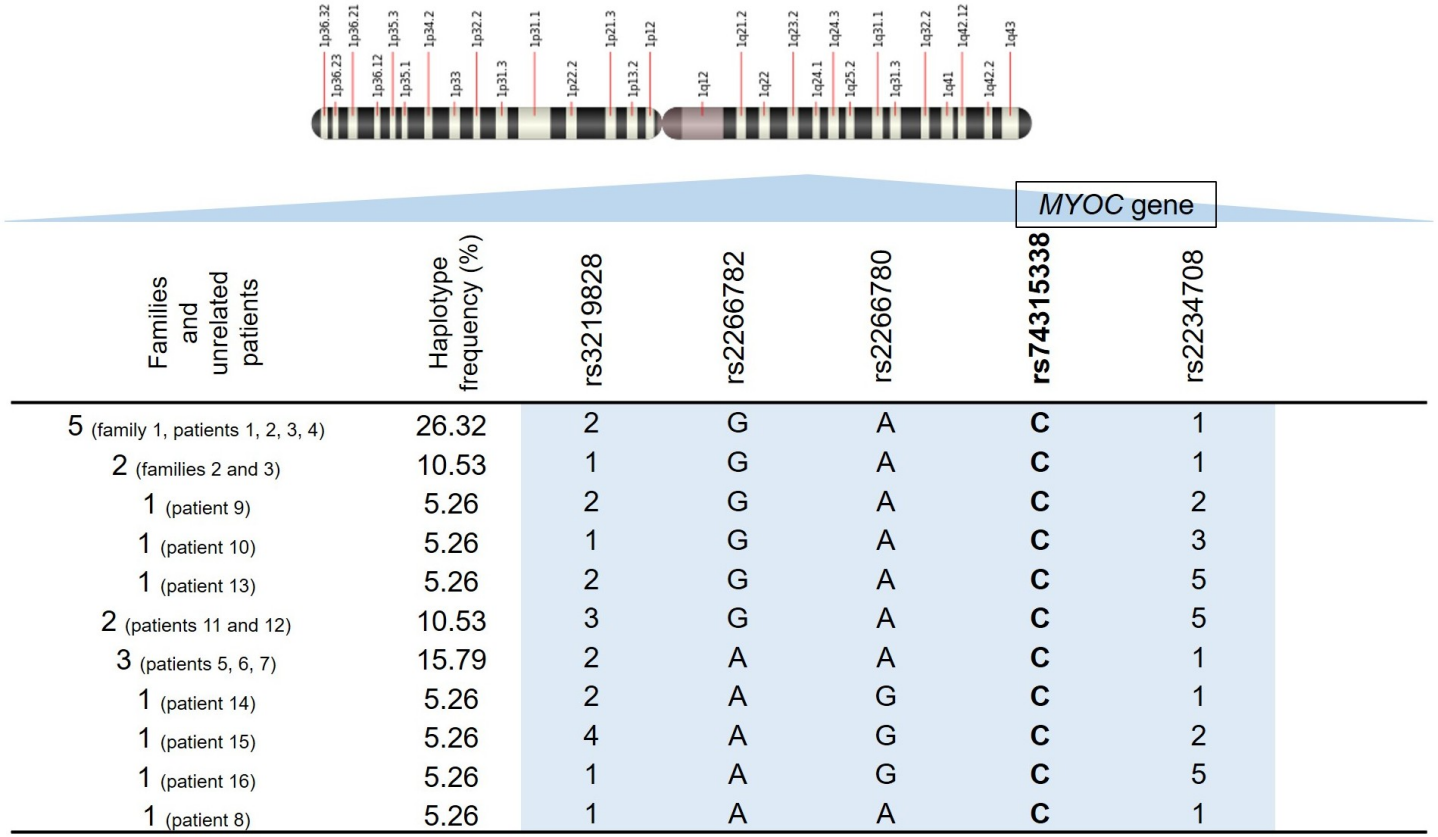
Representation of the haplotypes determined for individuals with the p.Cys433Arg variant at chromosome region 1q24.3. Haplotypes are formed by the markers rs3219828 (STR), rs2266782 (SNP), rs2266780 (SNP) and rs2234708 (STR). Each number represents a different allele in each microsatellite marker, as the letters represent different alleles in each SNP. The rs74315338, highlighted in bold, corresponds to the variant of interest. The first column indicates the number of each haplotype constructed and corresponding proband identity for each haplotype (family and unrelated patients). The position of the *MYOC* gene in relation to the markers is represented.

The haplotypes associated with the p.Cys433Arg variant were formed by the markers rs3219828 (STR), rs2266782 (SNP), rs2266780 (SNP) and rs2234708 (STR) (Fig 2). The haplotypes determined for individuals in the control group are in S1 Fig. In this group, we found a greater variation of haplotypes when compared to the group that harbors the variant, with the presence of alleles not observed in the haplotypes of the case group.

**Fig 3:**
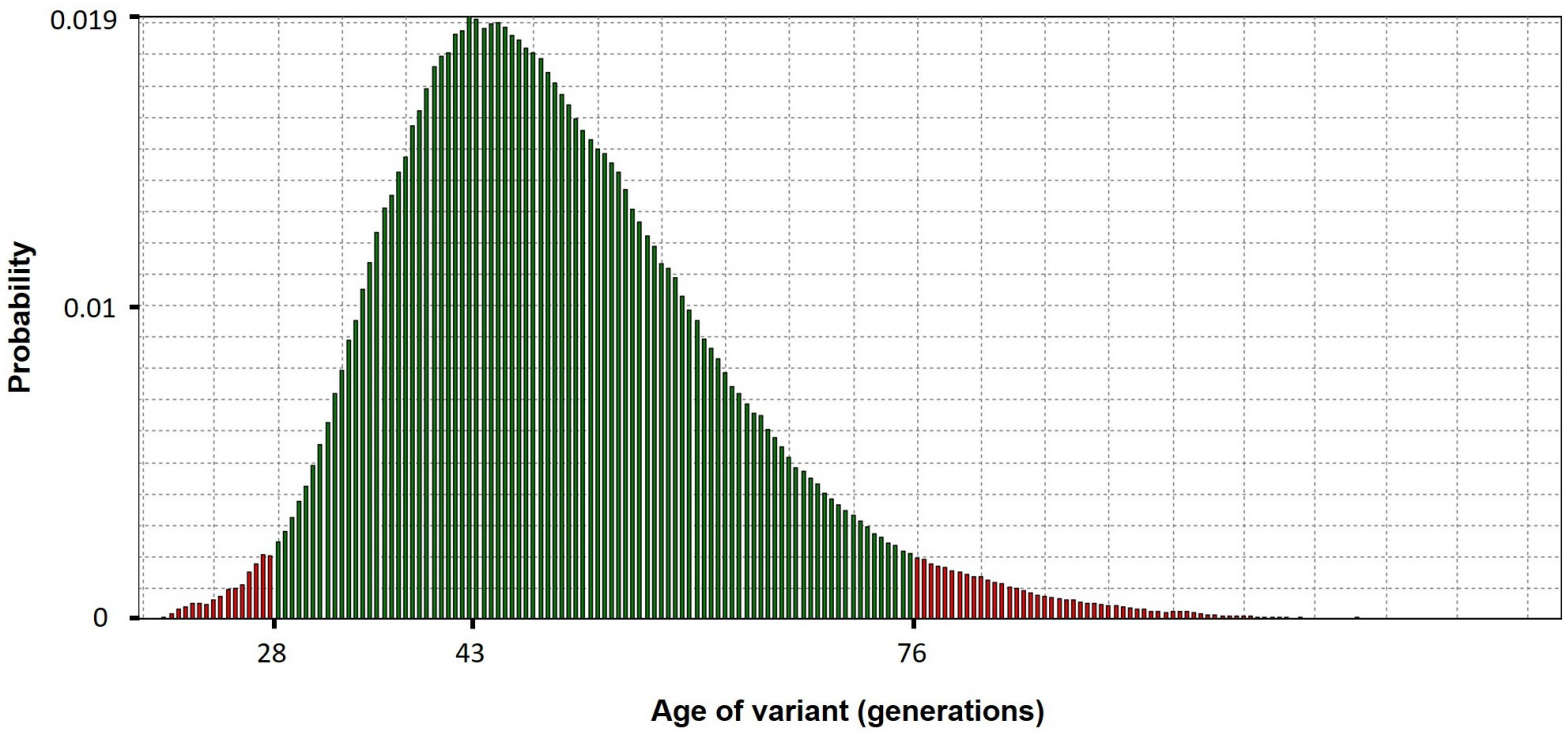
Age estimation for the p.Cys433Arg variant as assessed by DMLE+2.3. Histogram representing the age of the p.Cys433Arg variant in generations according to the probability of each age to occur, resulting from the iterations performed by the DMLE+ 2.3 software. Green bars indicate 95% CIs.

**Fig 4:**
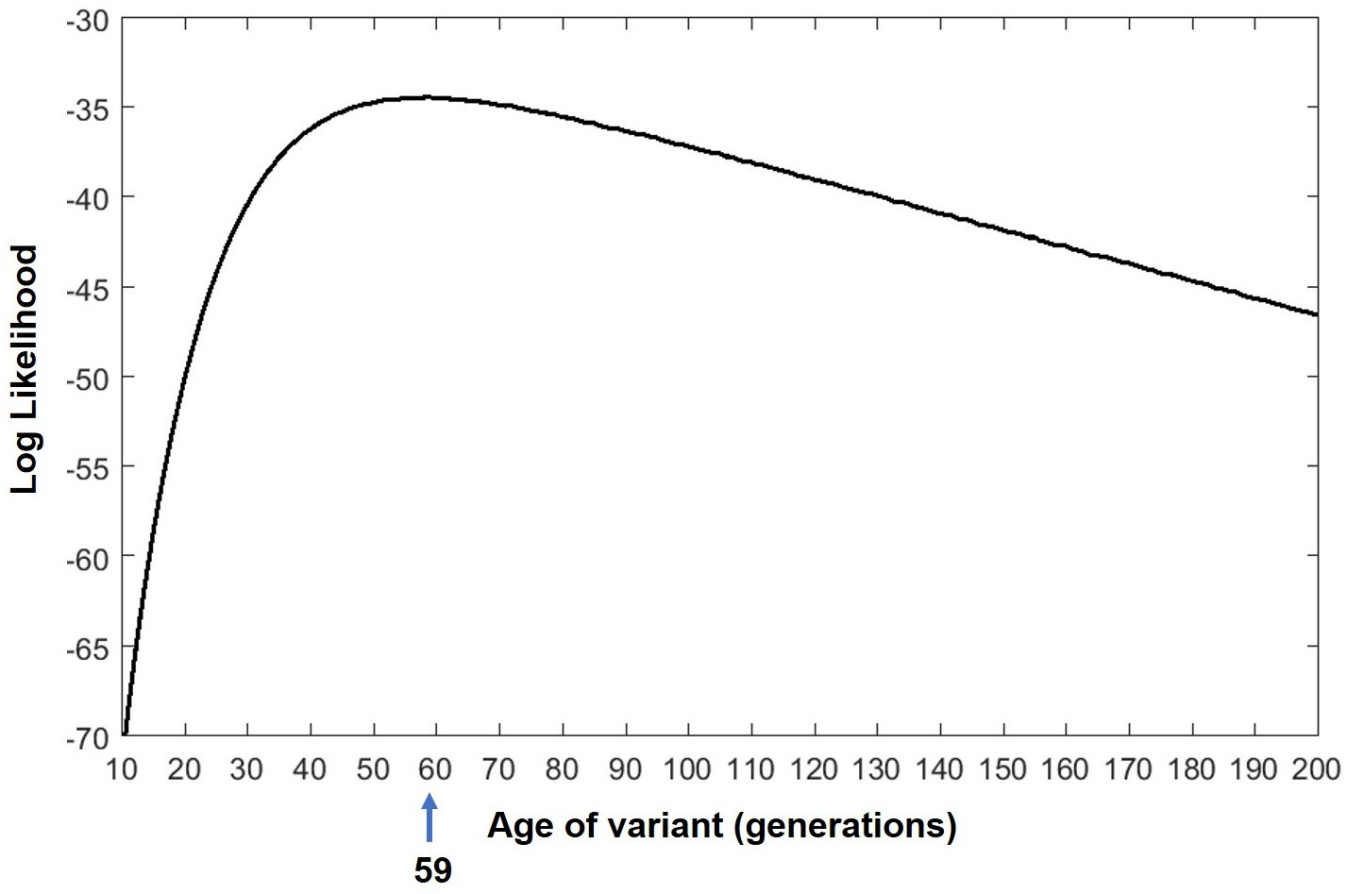
Age estimation for the p.Cys433Arg variant as assessed by BDMC21. Distribution of likelihoods of the p.Cys433Arg variant age (in generations).

Analysis performed by the DMLE+ 2.3 and BDMC21 softwares estimated that the age of the *MYOC* p.Cys433Arg variant might be 43 generations (95% CI: 28–76) (Fig 3) and 59 generations (95% CI: 40–100) (Fig 4), respectively. If the time interval between one generation and the next is considered to be 20 years [27,29, 35–37], the age of the variant lies in a range of approximately 560 to 1520 years (mode = 860 years) by DMLE+2.3 and approximately 800 to 2000 years (mode = 1180 years) by BDMC21. The application of the Luria-Delbrüch correction, resulted in g_0_ = 4.25, which means approximately four more generations than the numbers reached using the softwares for the age prediction of the variant. This correction lies within the confidence interval determined in the calculation of the age of the variant.

We also calculated the number of mutant chromosomes in the population of the Southeastern region of Brazil, resulting in approximately 484 chromosomes with the variant in this region of the country.

## Discussion

Founder variants are variants “observed with high frequency in a group that is or was geographically or culturally isolated, in which one or more of the ancestors was a carrier of the altered gene” (https://www.cancer.gov/publications/dictionaries/genetics-dictionary?cdrid=570712). Founder variants are important for studies in human genetics. Whenever these variants are discovered, they may help the molecular diagnosis of diseases, as part of an initial screening, which is cheaper than other genomic screening techniques [38]. Genetic tests for one or more founder variants are considered to be more efficient than for other rare variants [39–42]. Founder variants exhibit linkage disequilibrium (LD) with nearby genetic markers and with the use of LD mapping they can be helpful in the identification of the related genes. The size of the LD interval correlates inversely with the time since the variant occurred in the population and the identification of the same founder variant in different populations supports studies of the relationship among them [41,43].

A number of founder variants was detected in the *MYOC* gene, as in patients from Spain [44], where the heterozygous A>C transversion in codon 380 was detected in all affected members with JOAG from five families and was not detected in any of the 100 individuals without the disease. To determine whether a founder effect occurred, four microsatellite markers comprising the *MYOC* gene were analyzed. The results showed that a common haplotype associated with the disease was found in three families, indicating that the members of these families inherited the variant from a common ancestor. There are reports about founder variants in the *MYOC* gene in populations from France [45], Quebec [46] and Australia [47, 48]. These variants may have arisen within the population itself or may have been brought to the population immigrants [41]. In this case, immediately after the founder event, the allele could be found at higher frequency than it was before and could reach even higher frequency because of the strong genetic drift that might have occurred while the population was still small [49]. In our study we suggest a founder effect of the p.Cys433Arg variant, supporting the study of Vasconcellos et al [17].

As mentioned before, the p.Cys433Arg variant was reported exclusively in Brazil. We have suggested that this variant is associated with one common haplotype in individuals carrying the variant, and therefore it would have been inherited from a common ancestor [17]. Estimating the age of the p.Cys433Arg variant could help to understand the demographic history of this variant in our population and the projection of its prevalence and distribution in the Brazilian population. We estimate that about 484 people in the Southeast region of Brazil carry this variant. However, this estimate cannot be expanded to the whole country, because the presence of this variant has not been investigated in individuals from other Brazilian states.

The Brazilian population is the fifth largest population in the world with a high degree of miscegenation among native indigenous and immigrants. It is known that more than 2 million natives of several ethnic groups inhabited the territory before the arrival of the immigrants [50]. Colonization of the Brazilian territory by Europeans began about 500 years ago. During the early colonization history, approximately 500 thousand Portuguese individuals arrived in Brazil, followed by more than 4 million European immigrants in subsequent immigration waves, including more Portugueses, Germans, Italians and Spaniards. Meanwhile, during the slavery period (from the 16^th^ to 19^th^ century), approximately 4 million Africans from different regions of Africa were brought to the country [50–52].

Our study estimated the age of the p.Cys433Arg variant between 43 (860 years) and 59 (1180 years) generations. The estimates were obtained with two computational approaches, using DMLE+ 2.3 (43 generations) and BDMC21 (59 generations). We know that age-based variant analysis methods coalescent-based (DMLE+ 2.3) and likelihood-based (BDMC21) are quite efficient for young variants with fast population growth rate, which are the cases of the p.Cys433Arg variant and the Brazilian population, respectively [53]. The study of Clendenning et al (2008) [54], showed that the coalescent intra-allelic model to assess LD used by DMLE+ 2.3 software was more efficient to date the origin of the study variant than the method that gives a separate age estimate for each marker. The age of the variant was already known and the result obtained by DMLE was very accurate. Therefore, we believe that DMLE+ 2.3 software was the best choice to identify the age of the variant in this case. Besides, we defined the haplotype structure from the segregation analysis in the three families and also used statistically inferred haplotypes, analyzing a relatively small group of non-related individuals. Naturally sampling variation in allele frequencies can occur in the studies of rare variants and this inevitably will produce variance in LD estimations. Different marker types also tend to influence the estimates.

These results bring us to the beginning of the European colonization or even before that. Naturally, the question is whether the variant has arisen in the native population that inhabited Brazil or if it was brought by immigrants. The alternative about the appearance of this variant in native Brazilians is uncertain, since there are no studies directly addressing the incidence of glaucoma in the Brazilian indigenous population. A review of the ocular health of Brazilian natives has identified studies on indigenous peoples from the Amazon basin [55]. However, most of the reported cases were primary angle-closure glaucoma, and we know that the p.Cys433Arg variant is associated with primary open-angle glaucoma. Notwithstanding, it is important to notice that there are no studies investigating indigenous groups from the Southeast Brazil, where the p.Cys433Arg variant was detected. This fact, in addition to the absence of this variant in other studies around the world, does not exclude its origin among Brazilian native indigenous from the Southeast region.

The alternative hypothesis includes the arrival of the variant with the settlers. It could have arrived in Brazil with the slave trade among Africans but, if it was the case, it should have resulted in a younger variant with a lower number of generations compared to what we observed. In the present study, we analyzed a relatively small number of markers. The rs6133 SNP was the only marker that can be considered as an “ancestry marker”, because it has prominently different allele frequencies in European and African populations. Information from the Ensembl database (http://www.ensembl.org/index.html) shows that the C allele is more frequent in populations from South and East Asia (98% and 100%, respectively), Europe (88%) and America (87%), while in the African population the A allele is the most frequent (66%). We found the C allele as the most prevalent in our study sample. Since this, is the least frequent allele in the African population, it suggests that the variant may not have arrived in Brazil with the Africans.

Another possibility is the arrival of the variant with European colonizers. This is supported by the confidence interval of the age of the variant, which is close to the time of the arrival of Europeans in Brazil, when a founder effect could have occurred, as suggested by Vasconcellos et al [17]. The p.Cys433Arg variant may have come with European colonizers to Brazil, being extremely rare in Europe or even unique among settlers. Upon arrival in Brazil, its frequency could have decreased until nearly disappearing or it could have increased, as is the case in the Brazilian population. Noteworthy is the fact that this variant has never been described in European populations, which have been extensively studied.

The Brazilian population is highly heterogeneous, so the most accurate way to determine the ethnicity of the studied individuals would be with the use of ancestry markers or high density marker panels, an analysis that was not in the scope of this study. However, we know that Brazilians are the result of a number of migration events that were accompanied by intensive mixing of three main ancestral roots: Amerindians, Europeans and African [56]. In the Southeast region of Brazil, the European origin prevails, but with continuous migration events, the intermarriage between individuals of different ancestry is frequent [57]. A study conducted in our laboratory evaluated a group of 145 individuals from the same region of Brazil (São Paulo State), using high density array markers and found 62.4% European, 27.3% African and 10.2% Amerindian ancestry; however, the analyzed cohort was not preselected by ethnicity or skin color (personal communication).

Technological advances facilitating molecular genetic testing and overall request of societies for the preventing health care draw attention to the population-based screening for adult-onset disorders. The screening tests have a different purpose when compared to the diagnostic tests and aim to identify individuals who are more likely to have diseases. This may help to prevent the development of a serious illness or alleviate the severity of the disease. Knowledge of population-specific variants is necessary to compose population screening programs and proposes population-based intensive genetic studies.

The investigation about the demographics and age of the p.Cys433Arg variant, reported only in the Brazilian population, suggests a founder effect and its origin in native indigenous from the Southeast of Brazil or its arrival with European colonizers. The estimated number of about 484 people in Southeast Brazil carrying the variant contributes to our understanding of the distribution of JOAG in this region and reinforces the importance of screening JOAG suspects for this variant in the *MYOC* gene and may help to optimize screening tests for this population at risk.

## Supporting information

S1 Table Physical distance among markers and the *MYOC* gene. Location of *MYOC* gene: 1: 171,635,417–171,652,683.
(PDF)

S2 Table Table summarizing JOAG families and unrelated patients genotyping data. Determined genotypes of families and unrelated patients affected by JOAG. Each column represents a marker and each line represents an individual (in the case of unrelated patients) or each family. Each number (in case of microsatellites) represents a different allele from each marker. These numbers were assigned in ascending order according to the alleles found in each sample. The markers are sorted according to the position on the chromosome. x represents an undefined allele.
(PDF)

S1 Fig Representation of the haplotypes determined for control group. Haplotypes formed by the same markers as in the mutated group. Each number represents a different allele in each microsatellite marker, as the letters represent different alleles in each SNP. The rs74315338 highlighted in bold corresponds to the variant of interest. The first column indicates the amount of each haplotype constructed. The position of the *MYOC* gene in relation to the markers is represented.
(PDF)
