# Supplementary material for: Estimating the age of the p.Cys433Arg variant in the MYOC gene in patients with primary open-angle glaucoma
Source: PLoS One. 2018 Nov 16;13(11):e0207409. doi: 10.1371/journal.pone.0207409 (PMC6239314; doi:10.1371/journal.pone.0207409)
Supplement: S1 Table — Location of MYOC gene: 1: 171,635,417–171,652,683. (PDF) [file pone.0207409.s001.pdf]

Marker	Physical Distance among markers (Kb)	Physical Distance among *MYOC* gene (Kb)
rs6133	0	2070071
rs3221612	751618	1318451
rs3219828	543981	774468
rs2266782	216017	558451
rs2266780	6276	552175
rs2234708	16782	13350
D1S2815	92852	62236
D1S1165	86030	148599
rs3223566	1222531	1371601
rs3220994	948238	2319841
rs3220452	399822	2719665
rs3219958	130923	2850590

← ***MYOC* gene**
