# Supplementary material for: Estimating the age of the p.Cys433Arg variant in the MYOC gene in patients with primary open-angle glaucoma
Source: PLoS One. 2018 Nov 16;13(11):e0207409. doi: 10.1371/journal.pone.0207409 (PMC6239314; doi:10.1371/journal.pone.0207409)
Supplement: S2 Table — Determined genotypes of families and unrelated patients affected by JOAG. Each column represents a marker and each line represents an individual (in the case of unrelated patients) or each family. Each number (in case of microsatellites) represents a different allele from each marker. These numbers were assigned in ascending order according to the alleles found in each sample. The markers are sorted according to the position on the chromosome. x represents an undefined allele. (PDF) [file pone.0207409.s002.pdf]

Haplotypes – Families													
**Number**	**rs6133**	**rs3221612**	**rs3219828**	**rs2266782**	**rs2266780**	**rs74315338**	**rs2234708**	**D1S2815**	**D1S1165**	**rs3223566**	**rs3220994**	**rs3220452**	**rs3219958**
**1**	C	1	2	G	A	**C**	1	1	1	1	1	2	1
**2**	C	1	1	G	A	**C**	1	1	1	1	1	1	1
**3**	C	2	1	G	A	**C**	1	1	1	1	1	1	1
**Haplotypes – Unrelated patients**													
**1**	C	1	2	G	A	**C**	1	5	5	2	1	2	1
**2**	C	1	2	G	A	**C**	1	4	1	3	1	1	3
**3**	C	7	2	G	A	**C**	1	1	1	2	1	1	4
**4**	C	1	2	G	A	**C**	1	6	6	5	1	5	1
**5**	C	1	2	A	A	**C**	1	x	3	x	1	1	1
**6**	C	4	2	A	A	**C**	1	3	3	4	4	1	6
**7**	C	1	2	A	A	**C**	1	3	3	x	3	1	5
**8**	C	8	1	A	A	**C**	1	4	4	1	1	2	1
**9**	C	1	2	G	A	**C**	2	6	5	2	1	2	1
**10**	C	3	1	G	A	**C**	3	4	1	3	1	2	1
**11**	C	7	3	G	A	**C**	5	3	4	4	1	6	5
**12**	C	3	3	G	A	**C**	5	1	3	3	2	2	2
**13**	C	7	2	G	A	**C**	5	5	1	4	4	6	1
**14**	C	8	2	A	G	**C**	1	x	6	x	1	3	3
**15**	C	1	4	A	G	**C**	2	6	2	2	1	2	1
**16**	C	5	1	A	G	**C**	5	6	2	5	4	5	1
