# Supplementary material for: Estimating the age of the p.Cys433Arg variant in the MYOC gene in patients with primary open-angle glaucoma
Source: PLoS One. 2018 Nov 16;13(11):e0207409. doi: 10.1371/journal.pone.0207409 (PMC6239314; doi:10.1371/journal.pone.0207409)
Supplement: S1 Fig — Haplotypes formed by the same markers as in the mutated group. Each number represents a different allele in each microsatellite marker, as the letters represent different alleles in each SNP. The rs74315338 highlighted in bold corresponds to the variant of interest. The first column indicates the amount of each haplotype constructed. The position of the MYOC gene in relation to the markers is represented. (PDF) [file pone.0207409.s003.pdf]

Number of individuals with each haplotype	Haplotype frequency (%)	rs3219828	rs2266782	rs2266780	*MYOC* gene<br>**rs74315338**	rs2234708
6	9.23	4	A	A	**T**	2
3	4.61	2	A	A	**T**	3
2	3.08	2	A	A	**T**	5
1	1.54	4	A	A	**T**	6
1	1.54	2	A	A	**T**	1
1	1.54	5	A	A	**T**	7
1	1.54	5	A	A	**T**	2
1	1.54	4	A	A	**T**	3
5	7.69	4	A	A	**T**	4
4	6.15	3	A	A	**T**	4
3	4.61	2	A	A	**T**	4
1	1.54	1	A	A	**T**	4
3	4.61	1	G	A	**T**	4
1	1.54	5	G	A	**T**	4
4	6.15	2	G	A	**T**	4
6	9.23	1	G	A	**T**	1
4	6.15	2	G	A	**T**	3
7	10.77	4	G	A	**T**	2
1	1.54	5	G	A	**T**	6
1	1.54	3	G	A	**T**	3
1	1.54	5	G	A	**T**	5
1	1.54	2	G	A	**T**	1
1	1.54	3	G	A	**T**	2
1	1.54	2	G	A	**T**	2
1	1.54	6	G	A	**T**	2
1	1.54	4	G	A	**T**	6
1	1.54	4	G	G	**T**	2
1	1.54	4	A	G	**T**	3
1	1.54	3	A	G	**T**	3
